# Viral hepatitis and liver disease in the Mongolian community in London ('MoLo'): A mixed methods study protocol to investigate the epidemiology and burden of disease to inform clinical and public health interventions

**DOI:** 10.12688/wellcomeopenres.25134.1

**Published:** 2026-01-23

**Authors:** Emily Martyn, Nara Dashdorj, Jessica Carter, Indrajit Ghosh, Enkh-Oyun Amarbayasgalan, Khadija Said Mohammed, Elizabeth Waddilove, Marion Delphin, Kathryn Jack, Camila Picchio, Joy Ko, Michelle Berkeley, Rubin Rose-Key, Eleni Nastouli, Sema Mandal, Monica Desai, Sally Hargreaves, Douglas Gray, Jennifer Hay, James MacRae, Stuart Flanagan, Julian Surey, Philippa C. Matthews

**Affiliations:** 1 The Francis Crick Institute, London, England, UK; 2 Division of Infection and Immunity, University College London Faculty of Medical Sciences, London, England, UK; 3 Onom Foundation, Ulaanbaatar, Ulaanbaatar, Mongolia; 4 Migrant Health Research Group, Institute for Infection and Immunity, City St George's University of London, London, UK; 5 Queen Mary's Univesity of London, Wolfson Institute of Population Health, London, England, UK; 6 Hepatitis Service, Mortimer Market Centre, Central and North West London NHS Foundation Trust, London, England, UK; 7 Mongolian Community Organisation, London, UK; 8 Division of Biosciences, University College London Faculty of Life Sciences, London, England, UK; 9 Hepatology Department, Nottingham University Hospitals NHS Trust, Nottingham, England, UK; 10 University of Barcelona, Barcelona Institute for Global Health, Barcelona, Spain; 11 Division of Infection, University College London Hospitals NHS Foundation Trust, London, England, UK; 12 Department of Infection, Immunity and Inflammation, University College London, Great Ormond Street Institute of Child Health, London, England, UK; 13 Department of Virology, University College London Hospitals NHS Foundation Trust, London, England, UK; 14 Blood Safety, Hepatitis, STI & HIV Division, UK Health Security Agency, London, UK; 15 Institute for Global Health, University College London Faculty of Population Health Sciences, London, England, UK

**Keywords:** HBV, HCV, HDV, HIV, blood borne virus, Mongolia, Inclusion Health, screening, epidemiology, MASLD, liver health, Community engagement, implementation research, mixed-methods, qualitative

## Abstract

**Background:**

Chronic viral hepatitis infections (hepatitis B (HBV), C (HCV) and D viruses (HDV)) are responsible for over 1 million deaths annually, due to cirrhosis and liver cancer. Mongolia has a high prevalence of all these infections, resulting in the highest incidence and mortality from liver cancer in the world. Other factors, such as metabolic dysfunction-associated steatotic liver disease (MASLD) can impact viral hepatitis, although the interaction is not fully understood. Several successful viral hepatitis screening programmes have been carried out among Mongolians living in Spain, USA, and Sweden.

**Protocol:**

We describe a community-informed protocol for the implementation of liver health screening among Mongolians living in London (UK), designed by a multi-disciplinary team. This observational, mixed-methods study (‘Hep-MoLo’) has three domains. (i) In the clinical domain, liver screening events will be held in London, in collaboration with the Mongolian Community Organisation. An awareness-raising educational component will precede point-of-care screening for blood-borne infections (HBV, HCV, HIV), liver fibrosis and steatosis, and screening for cardiometabolic risk factors (obesity, hypertension, dyslipidaemia, diabetes); (ii) Laboratory studies will focus on the interaction between HBV and MASLD; (iii) A qualitative approach will be used to explore community views on liver health screening, access and engaging in care.

**Discussion:**

This protocol provides a framework for a public health intervention targeting a high-risk population, combined with laboratory and qualitative research to give a multi-dimensional insight into viral hepatitis and liver health in the London Mongolian community. This is a community-academic-clinical partnership, fostering collaboration to generate data to inform clinical and public health interventions.

## Plain language summary

Liver disease is the 11th leading cause of death worldwide, due to liver scarring (cirrhosis) and liver cancer. Two major liver disease causes are long-term viral hepatitis infections (caused by hepatitis B virus (HBV), hepatitis C virus (HCV) and hepatitis D virus (HDV)), and liver stress caused by conditions such as too much liver fat, high blood pressure, diabetes, obesity and/or abnormal blood fats. The exact relationship between all these factors on liver disease is not yet fully understood.

This protocol describes a liver health study among Mongolians living in London, since Mongolia has very high rates of viral hepatitis and liver disease. Clinical outreach teams have joined a Mongolian Charity and Community Organisation to plan community-based liver screening events.

Researchers will use clinical information and blood samples to explore the interaction between factors that cause liver disease. They will measure fats (lipids), immune markers (cytokines) and sequence the genetic code of both the human and hepatitis B virus. Researchers will also talk with people from the Mongolian community to understand community views on viral hepatitis screening and access to healthcare.

This study will develop a clinical-academic-community partnership, set the scene for future studies, and improve access to healthcare and liver-health awareness.

## List of Abbreviations

**Table:** 

Abbreviation	Meaning
BBV	Blood Borne Virus
CAP	Controlled Attenuation Parameter
CNWL	Central North West London NHS Trust
DAA	Direct Acting Antiviral
DSH	Data Safe Haven
EASL	European Association for the Study of the Liver
EPR	Electronic Patient Record
GP	General Practitioner (primary health care doctor)
HbA1c	Glycated haemoglobin
HBcrAg	Hepatitis B Core-Related Antigen
HBsAg	Hepatitis B Surface Antigen
HBV	Hepatitis B Virus
HCV	Hepatitis C Virus
HDV	Hepatitis D Virus
HIV	Human Immunodeficiency Virus
HLA	Human Leukocyte Antigen
ICF	Informed Consent Form
LFTs	Liver Function Tests
MASLD	Metabolic Dysfunction-Associated Steatotic Liver Disease
MCO	Mongolian Community Organisation
MMC	Mortimer Market Centre
NICE	The National Institute for Health and Care Excellence
NHS	National Health Service
PLWH	People living with HIV
PLWHB	People living with HBV
POCT	Point Of Care Test
PPIE	Patient and Public Involvement and Engagement
TE	Transient Elastography
UCL(H)	University College London (Hospitals)
VCTE	Vibration Controlled Transient Elastography
WHO	World Health Organization

## Introduction

**Figure 1.**
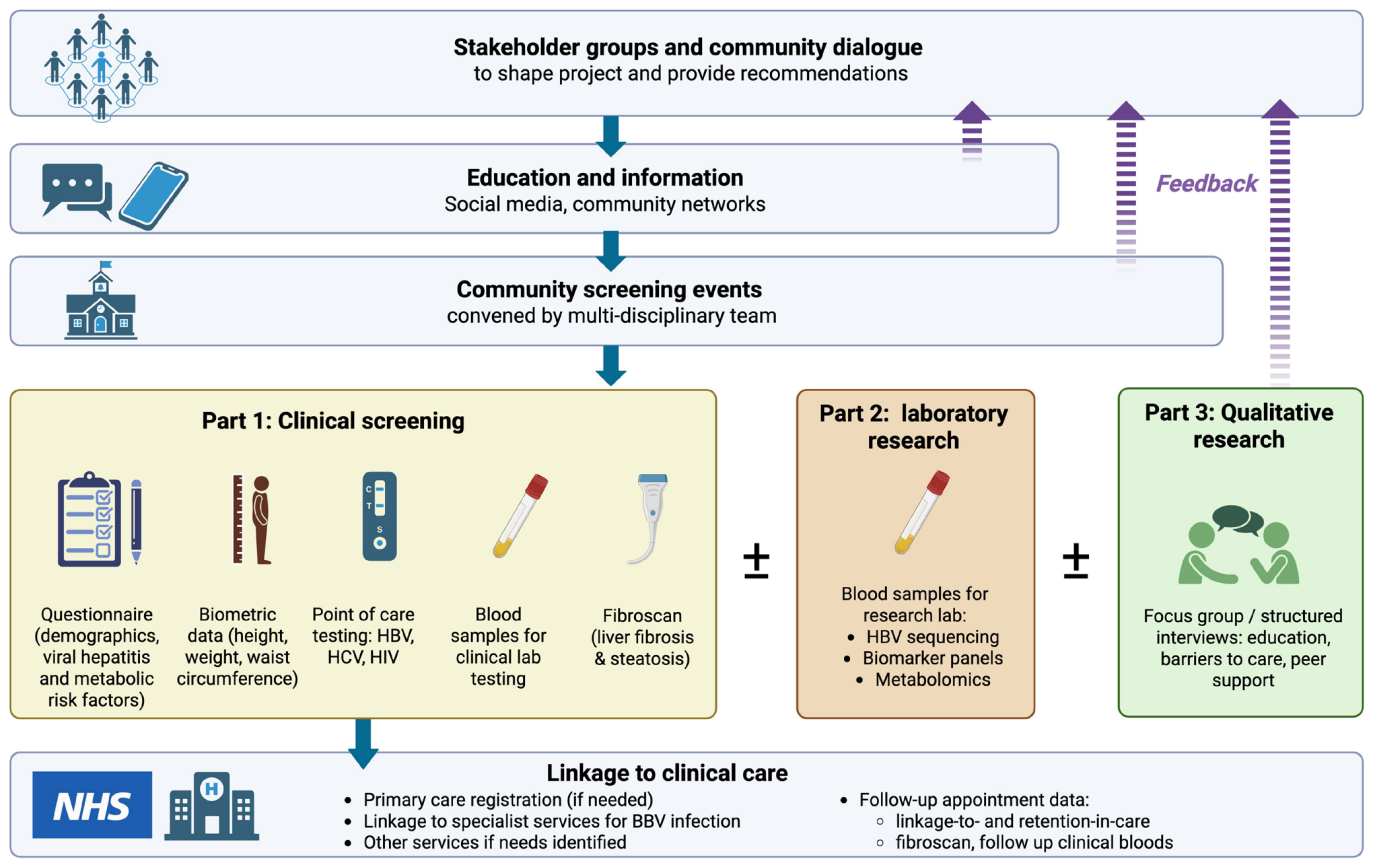
Viral hepatitis in the Mongolian Community in London: An investigation of epidemiology and burden of disease to inform clinical and public health interventions (Hep-MoLo) schematic describing study workflow. Overarching community engagement and ongoing feedback informs all other aspects of the project. Education and social media use are guided by our collaborators: Mongolian Community Organisation and the Onom Foundation. There are 3 project domains: clinical, laboratory and qualitative research. Created with BioRender.com *Abbreviations: HBV, hepatitis B virus; HCV, hepatitis C virus; HIV, human immunodeficiency virus; BBV, blood borne virus*.

Chronic liver disease is a major global health problem, responsible for 2 million deaths annually due to liver cirrhosis and hepatocellular carcinoma ^1^. Mongolia has the highest incidence and mortality rates from liver cancer globally, with 85.6 new cases per 100,000 people in 2020 ^2^. Two important contributors to the burden of global liver disease are chronic viral hepatitis, affecting an estimated 304 million people, and metabolic-dysfunction associated steatotic liver disease (MASLD), affecting approximately 1.6 billion people worldwide ^3, 4^. We here present a protocol for a pilot mixed methods study titled “Viral hepatitis in the Mongolian community in London: An investigation of epidemiology and burden of disease to inform clinical and public health interventions (Hep-MoLo)”, to address liver disease in the London Mongolian community ( Figure 1).

### (i) Viral hepatitis - global landscape

Chronic viral hepatitis is mainly caused by infection with hepatitis B virus (HBV), hepatitis C virus (HCV), and hepatitis D virus (HDV) via parenteral or vertical transmission ^5^. HDV can only occur in the context of HBV infection and causes accelerated liver disease ^6^. Despite there being no curative treatment for HBV, viral suppression can be achieved via nucleot(s)ide analogues (e.g. tenofovir). The 2024 World Health Organization (WHO) Global guidelines relaxed treatment criteria to make many more people treatment-eligible, therefore identifying people living with chronic hepatitis B (PLWHB) and linkage-to-care will be crucial to ensure those eligible receive treatment ^7^. There is a safe and effective preventative HBV vaccine, which has contributed to a global decline in HBV prevalence, but challenges in widespread, reliable access persist. A new treatment for HDV (bulevirtide) reduces viral load, liver inflammation and fibrosis and became available in the NHS in 2023 ^8^. Substantial progress has been achieved in the treatment of HCV, with safe and effective direct acting antivirals (DAAs) now available to cure infection within 8–12 weeks. Despite progress being made in the elimination of chronic viral hepatitis, it still causes 1.3 million deaths each year ^3^. We are not on track to meet international sustainable development goals (SDG) to reduce new infections by 90% and deaths by 65% by 2030 ^9^ (referred to as ‘SDG30’).

### (ii) Viral hepatitis - United Kingdom context

In line with SDG30, the UK government pledged to eliminate chronic viral hepatitis as a public health threat by 2030 ^10^. Considerable progress has been made towards SDG30 for HCV in England, driven by a national elimination programme focussing on case-finding, wide accessibility of DAAs, peer support and collaboration between NHS, peer support and third sector and industry ^11^. Despite the success of these programmes which lead to a 60% reduction in HCV prevalence and a ~40% reduction in HCV-associated mortality, further work is required to meet the SDG30 targets ^12^. In April 2022, opt-out blood borne virus screening (HIV, HBV and HCV) was introduced across 34 Emergency Departments in England. Over the first 33 months of the programme, there were more new HBV diagnoses (3,667 new cases, 879, 724 tests) than HCV (831 new cases, 1,060,035 tests) or HIV (719 new cases, 1,377,299 tests) diagnoses (data from 24 sites), highlighting undiagnosed HBV as a public health concern in England, requiring enhanced attention to achieve viral hepatitis elimination goals ^13^.

### (iii) Metabolic liver disease

**Figure 2.**
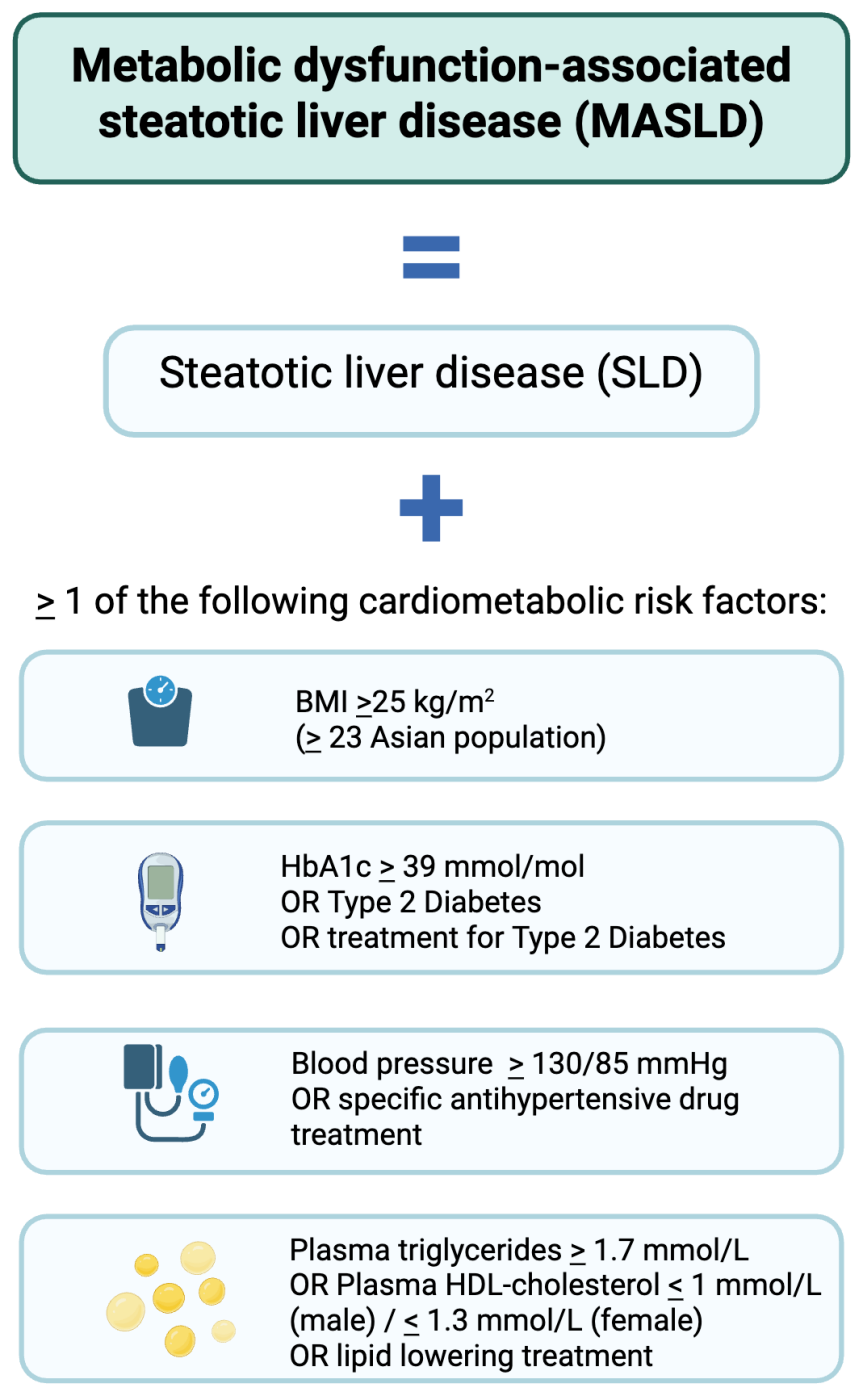
Metabolic dysfunction-associated steatotic liver disease (MASLD) definition schematic. Adapted from Rinella *et al.*, 2024 ^14^. Created with BioRender.com. *Abbreviations: BMI - Body Mass Index, HbA1c - haemoglobin A1c (glycosylated haemoglobin), HDL - high density lipoprotein.*

‘MASLD’ is defined as steatotic liver disease plus at least one of diabetes, hypertension, dyslipidaemia and/or overweight/obesity ( Figure 2) ^14^. It is the most common cause of liver disease, affecting 25–35% of the general adult population ^4^. The term MASLD was introduced in 2023 to replace non-alcoholic fatty liver disease (NAFLD), moving away from pejorative language, and recognising the importance of cardiometabolic factors in pathogenesis ^15^.

### (iv) High risk and underserved populations

There are substantial inequities in liver disease. People experiencing lower socio-economic and educational levels have the highest incidence of liver cancer and the worst liver disease care ^16^. For example, social determinants of health including barriers to accessing affordable, nutritious food, food literacy and physical inactivity contribute to increased MASLD prevalence in socially deprived populations ^17^. Chronic viral hepatitis disproportionately affects inclusion health populations, who experience multiple barriers to accessing healthcare ^18^. For example, migrant groups may experience compounding challenges when accessing healthcare including stigma, language barriers, socioeconomic disadvantage, and navigating unfamiliar healthcare systems ^18, 19^. Lessons from the national HCV elimination programme highlight the importance of holistic, empathetic, accessible and inclusive liver services, incorporating patient and public involvement and integration with other services (e.g. pharmacy, third sector) to reach those most affected and deliver equitable care ^20^.

### (v) Evidence gaps

As MASLD prevalence is rising in the context of increasing global prevalence of obesity and type 2 diabetes, the overlap with viral hepatitis is set to increase. The interplay between MASLD and viral hepatitis, in particular HBV, is not well understood ^21^ and is therefore an area of expanding interest for risk stratification. Moreover, evidence gaps exist in our understanding of why some PLWHB develop liver disease and some remain relatively unaffected throughout life ^22, 23^. Multiple factors may influence disease progression, for example, viral genetic sequence (10 HBV genotypes), host genetic sequence (Human Leukocyte Antigen (HLA) genes significantly impact viral immunity and some single nucleotide polymorphisms can increase risk of MASLD), and host immune and metabolic profiles ^22– 25^. Further knowledge on viral factors and host factors may help provide further insight into disease progression and risk stratification. From the public health perspective, enhanced focus on the best approaches to address liver care inequity is urgently required.

## Rationale

Mongolia has the highest age standardised incidence rate and mortality rate for liver cancer globally, reflecting the high prevalence of chronic viral hepatitis ^26– 28^. Our study focuses on the Mongolian population living in England, recognising the high liver disease risk of this group. There are approximately 4000 Mongolians in the UK, mostly concentrated in major cities, including London, Nottingham and Manchester ^29^. Previous viral hepatitis screening programmes offered to the Mongolian population living in Sweden, Spain and the United States (U.S.) indicate a high prevalence of chronic viral hepatitis among the Mongolian community: 3.6 – 9.7% HBV (hepatitis B surface antigen (HBsAg) positive), 0.9 – 3.9% HBV/HDV coinfection (anti-HDV positive) and 0.5 – 7.1% HCV (HCV RNA positive) ^30– 34^. In Sweden, researchers found HBsAg prevalence and HDV coinfection in the migrant cohort were significantly lower than an age-matched native Mongolian cohort (HBV: 4.4 vs 9.8%, HDV: 2.1 vs 5.1%) ^34^. A 2021 systematic review estimated that the age-adjusted chronic HCV infection prevalence in adults living in Mongolia was 6%, with 14% anti-HCV prevalence, higher than reported in Egypt (which is known as a highly endemic country) ^35^.

The prevalence of cardiometabolic risk factors is rising in Mongolia ^36, 37^. In 2019, 10% of adults over 30 years had type 2 diabetes in Mongolia, a threefold increase compared to 1999 ^36^. Obesity prevalence has increased from 7.5 to 14.4% in men and 12.5 to 20.4% in women between 2005 and 2019 ^37^. On a global scale, rising cardiometabolic risk factors are contributing to an increasing MASLD prevalence, but MASLD in the Mongolian population and the impact on outcomes in viral hepatitis remains under-studied ^4^.

We describe a protocol for the 'Hep-MoLo' study, designed to address these multi-factorial contributors to liver health in the Mongolian community. This study will focus on liver health screening in the Mongolian community in London, establishing clinical-academic-community collaboration to develop a pathway for diagnosis and clinical care into which we will embed translational research ( Figure 1). Within the public health and clinical element, we describe a liver health screening programme pilot. The research laboratory element will facilitate deeper understanding of host (e.g. immune, metabolic, genetic) and pathogen (viral genetic and other biomarkers) roles in viral hepatitis pathogenesis within a well characterised cohort. Finally, a qualitative element will give us insight into important factors which influence Mongolian migrants accessing liver healthcare in the UK.

## Governance

This protocol has been externally peer reviewed by two independent reviewers, received local institutional approval by University College London (UCL), University College London Hospital (UCLH) and the Francis Crick Institute Human Biology Facility, and has received external ethical approval from a NHS regional ethics committee (REC reference 25/LO/0126). It is included in the National Institute for Health and Care Research (NIHR) Research Delivery Network (RDN) portfolio (CPMS ID 64430).

## Protocol

### 1. Aim

The primary aims of this study are to gather evidence on the implementation of a screening programme and to estimate the prevalence of chronic viral hepatitis (HBV, HCV and HDV) and MASLD in the Mongolian community living in London to help inform public health intervention and treatment strategies.

### 2. Study design and objectives

This is an observational, mixed methods, cross sectional, implementation study, with three main domains and associated **primary objectives** ( Figure 1):

**Clinical screening domain (part 1):**

Among the London Mongolian community, we aim to:

- Develop community engagement and develop a stakeholder group to support the establishment of a screening and research programme for chronic viral hepatitis infection (HBV, HCV, HDV).
- Road-test a liver health community screening programme.
- Raise awareness of liver health, in particular chronic viral hepatitis (HBV, HCV and HDV) and MASLD.
- Estimate the prevalence of chronic viral hepatitis (HBV, HCV and/or HDV).
- Estimate the prevalence of MASLD and presence of cardiometabolic risk factors (obesity, hypertension, diabetes and/or dyslipidaemia).

**Laboratory research domain (part 2):**

Using data and samples collected from the London Mongolian community attending screening events, we will:

- Compare lipidomic profiles between four groups defined as follows:
  (1) PLWHB and MASLD,
  (2) PLWHB only,
  (3) people living with MASLD only,
  (4) “healthy controls” (without liver disease).
- Generate pilot data to investigate the associations between liver disease and demographic, clinical and laboratory factors (e.g. host immune and lipidomic profile, viral and host (HLA) sequencing), among people living with chronic viral hepatitis.

**Qualitative domain (part 3):**

Among the London Mongolian community engaging with this programme, we will set out to understand experiences, attitudes, facilitators, and barriers to:

- Testing for chronic viral hepatitis and cardiometabolic risk factors,
- Attending community healthcare screening events,
- Accessing prompt and sustained healthcare for liver disease e.g. chronic viral hepatitis.

Several **secondary objectives** are nested within the project. Among the London Mongolian community, we will:

- Use existing clinical pathways to connect individuals to appropriate local primary and secondary clinical services, namely a General Practitioner (GP) in their area of residence, clinical follow up for blood borne virus (BBV) infection (at Central and North London NHS foundation trust (CNWL) or a local hepatology service according to participant preference), and any other clinical services if other needs are identified.
- Estimate the prevalence of people living with HIV and/or syphilis.
- Evaluate linkage-to-care at 3 months and retention-in-care after 12 months among those diagnosed with viral hepatitis infection.

### 3. Public and Participant Involvement and Engagement (PPIE)

PPIE was central to informing study design. Members of the Mongolian community including both clinical practitioners, community organisations, and peer support workers with lived experience of hepatitis B are key members of the study team and have attended study design meetings and given feedback on the protocol and other study documents.

**Figure 3.**
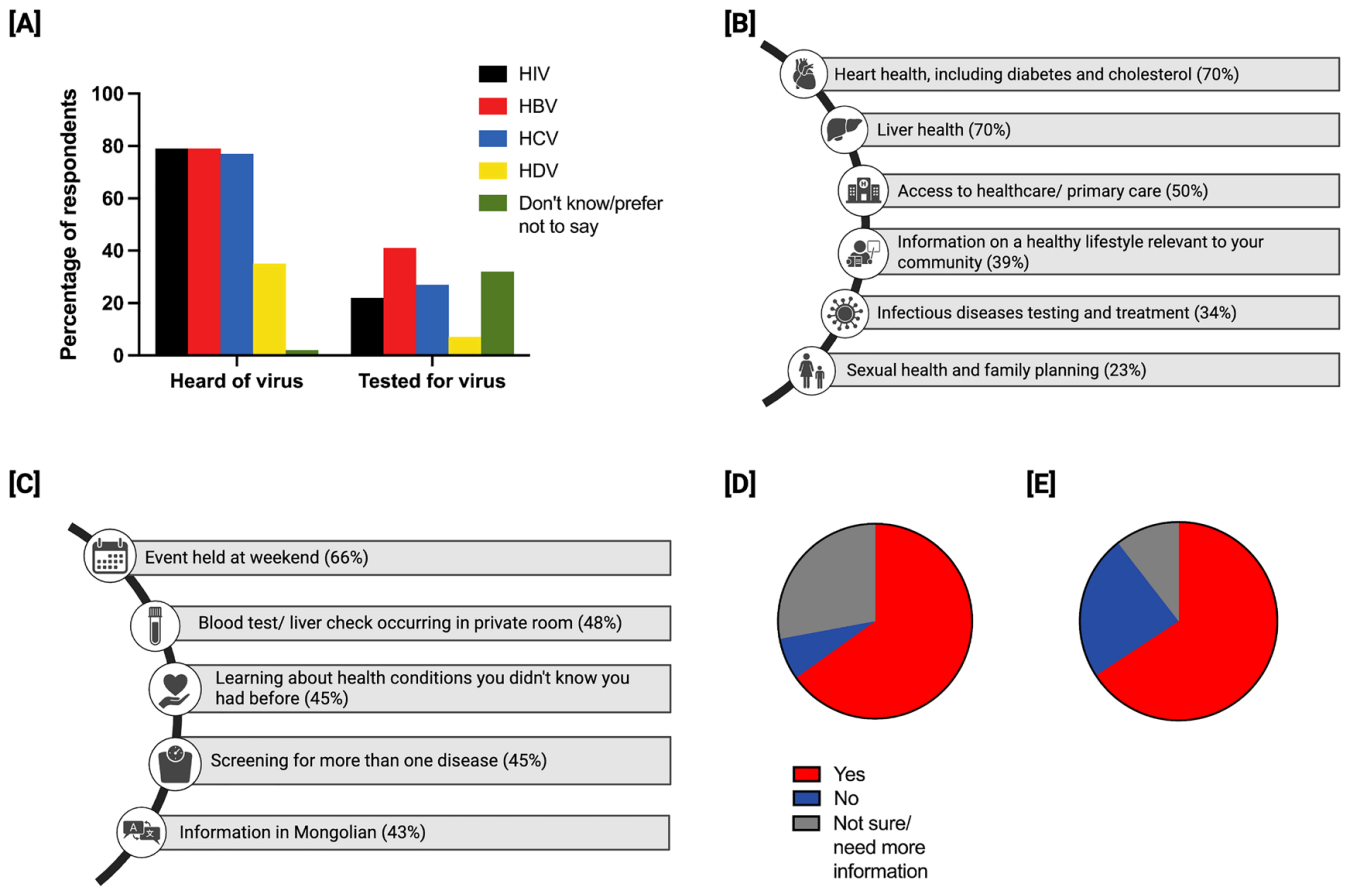
Hep-MoLo Participant and Public Involvement and Engagement. Key results from an online survey for the Mongolian community ^38^. There were 44 respondents. [ **A**] Blood borne virus awareness: respondents were asked to indicate if they had heard of the virus, and whether they had been tested before. [ **B**] Community health priorities: percentage of respondents who identified each statement as one of their top 3 health priorities. [ **C**] Facilitators for attending community screening events: percentage of respondents who identified each statement as a facilitating factor. Attitudes towards [ **D**] attending a liver screening event (yes 65%, not sure 28%, no 7%), [ **E**] consenting to take part in research (yes 68%, not sure 11%, no 20%). *Abbreviations: HIV - human immunodeficiency virus, HBV - hepatitis B virus, HCV - hepatitis C virus, HDV - hepatitis D virus.*

The study team designed and circulated an online survey to the Mongolian community between 1st March and 30th April 2024 to explore attitudes and awareness of viral hepatitis, liver screening and research involvement. The survey was distributed via social media, email, and word of mouth and results are available online ^38^. Forty-four people responded, 68% female and aged between 19 and 64. Whilst the response rate was limited, the majority of respondents felt that liver and cardiovascular health were community health priorities, valued screening for more than one disease and same-day results, were more likely to attend at weekends and would prefer information to be available in Mongolian ( Figure 3). The feedback influenced protocol design.

On 6th July 2025 the Hep-MoLo study team attended the London Naadam Festival, an important annual celebration of Mongolian culture, to raise awareness about the project. A report of PPIE activities from this event is available to view online ^39^. We will continue to consult with the Mongolian community as the events are implemented to gather feedback and adjust our approaches (within the terms of the study ethics and governance).

### 4. Study setting

This study is based upon a multi-disciplinary clinical-academic-community partnership. The team includes specialist nurses, doctors and peers support workers from Find & Treat (UCLH) and Mortimer Market Centre (MMC, part of CNWL). The Find & Treat team specialise in providing outreach infectious disease screening to people who experience multiple barriers to accessing healthcare, for example vulnerable migrants or people experiencing homelessness, also known as ‘inclusion health’ groups ^40^. MMC is a sexual health and HIV/hepatitis service, with close links to the Find & Treat team and specialist inclusion health clinicians ^41, 42^. The study is sponsored by UCL and the chief investigator is based at the Francis Crick Institute, a large biomedical research institute based in London ^43^. Other key team members include founders of two civil society organisations: Onom Foundation, addressing healthcare and education challenges among Mongolian people globally, and the Mongolian Community Organisation (MCO), a London based charity providing cultural events and education to the local Mongolian community ^44, 45^.

The Hep-MoLo team will host a series of outreach liver screening events, using the same venue as the MCO in London, which has key amenities, including separate rooms, wifi, computers, toilet facilities, and good public transport links. Find & Treat will also provide their outreach van creating additional space for study activities.

Clinical blood tests and research samples will be processed and analysed by a local clinically validated laboratory and the Francis Crick Institute, respectively. Qualitative study focus groups will be held at the MCO premises and interviews via telephone. Clinical follow up will be facilitated by the inclusion health teams (specialist doctors, nurses and peer support workers) via UCLH and CNWL NHS foundation trusts (see Section 6.2.2 for further details).

### 5. Study population

*5.1 Inclusion criteria*

We will invite Mongolian adults (aged 18+) (born in Mongolia or elsewhere, as long as they identify as belonging to the Mongolian community) to attend community viral hepatitis and liver health screening events.

*5.2 Exclusion criteria*

We will not recruit children or anyone who does not have the capacity to provide valid informed consent.

*5.3 Participant recruitment*

Prior to screening events, potential participants will be contacted with written information (available in Mongolian and in English) with study details, alongside an invitation to attend the screening event. The study will be advertised via a Mongolian community Facebook group and word of mouth and posters via the MCO. Participants will be asked to register interest by completing a secure Microsoft form, via a NHS one drive account, and the participant information sheet will be emailed or handed out in person in advance of the day. They will also be provided with a nhs.net study email address for further information about the project. We will also allow enrolment of participants who have not pre-registered, since there will be information available and time to consider on the day, and further screening opportunities at subsequent events.

### 6 Study procedures

*6.1 Consent*

Study enrolment will take place on the day of the screening event facilitated by a trained member of the clinical research team. Potential participants will be provided with written and verbal information and opportunity to ask questions ^46^. Consent will be electronic, but written consent forms will be available in the case a technical issue arises, or chosen based on participant preference. The consent forms will be in Mongolian and English, and an interpreter will be available to assist with the consent process ^46^.

Participants will be given the option for a three-tiered consent. Tier 1 involves clinical screening only; tier 2 gives consent for additional samples for laboratory research and tier 3 enables researchers to contact them later to take part in qualitative research. The qualitative research section has a separate consent form. It was necessary to have written consent, even for part 1 of the study which is in line with standard BBV team outreach screening practices, to enable full reporting write up, analysis and dissemination of the results, and therefore maximise translational outcomes and impact from the project.

*6.2 Clinical domain (part 1)*

Up to five community-based screening events will be held over a period of 6 months, predominantly on weekends, in line with community preference ( Figure 3). At each event, we will provide education and awareness-raising information about chronic viral hepatitis and liver health, available in written format (in English and Mongolian) and from face-to-face discussion, together with explanation of the purpose of the clinical and research activities.

Potential participants will be asked to avoid eating for 6–8 hours before the event as this will improve consistency of imaging and lipidomic data. This will be communicated to people who sign up in advance of the event and will be added to posters and promotional material. Information on fasting time will be captured in the questionnaire and lack of fasting will not be an exclusion criterion. Refreshments will be provided after the screening activities.

**6.2.1 Clinical assessment approach for all participants**

To enable clinical follow up, participants will first be registered on the UCLH electronic patient record (EPR), requiring name, date of birth and contact details (phone number, address). Participants will be asked for their NHS number and registered GP, however if they are unregistered and/or do not have legal status, this will not be a barrier to testing. The Find & Treat team will support participants with GP registration and accessing care as required. These details will remain in the clinical domain and will not be used for research.

**Figure 4.**
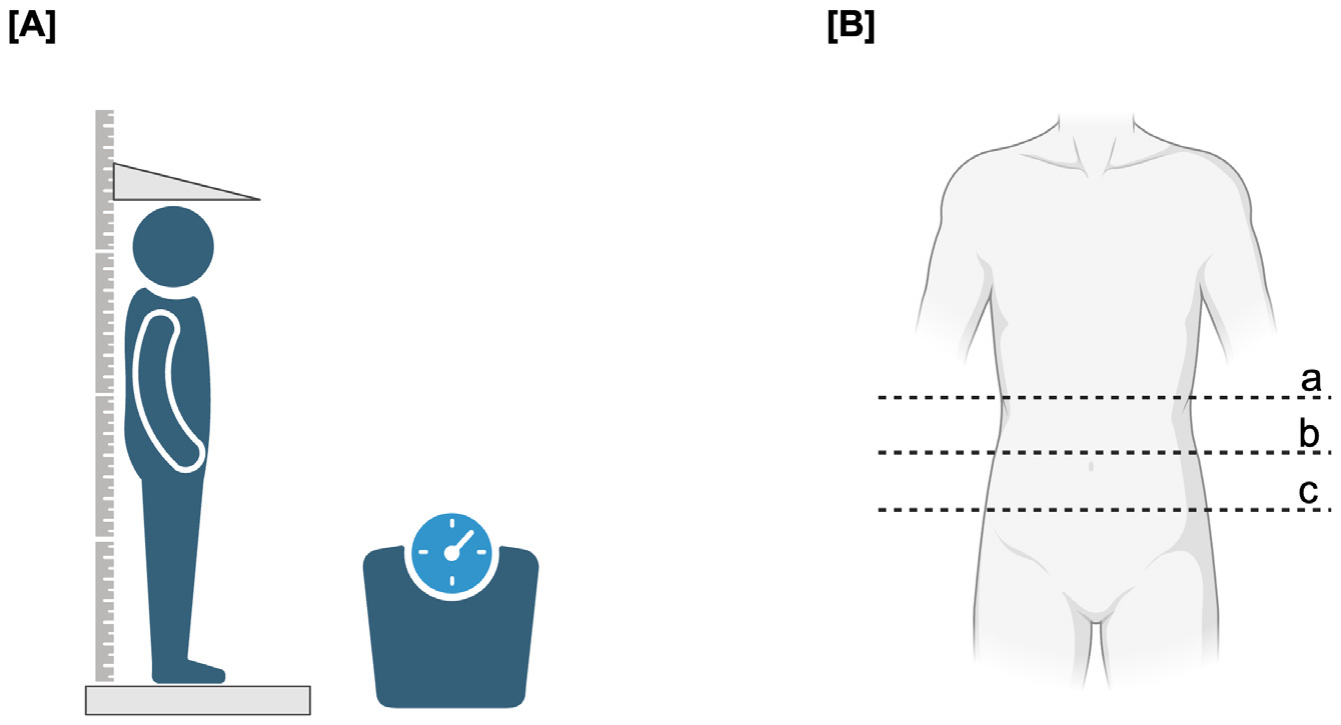
Methods of obtaining anthropometric measurements used in the Hep-MoLo project. [ **A**] Stadiometer to measure height and scales to measure weight. [ **B**] Waist circumference measurement: taken at the midpoint (b) between the lowest margin of the last palpable rib (a) and the top of the iliac crest (c) ^47^. Created with BioRender.com.

Following registration and informed consent, participants will be asked to complete a questionnaire to collect data about demographics, past medical history, drug and social history relevant to viral hepatitis and MASLD ^46^. Next, a series of anthropometric measurements and point-of-care tests (POCTs) will be performed ( Table 1, Figure 4).

**Table 1.** Point-of-care tests (POCT) being performed on liver outreach screening days in the Hep-MoLo project.

Data	POCT	Measurement (units)	Comments
Anthropometric data	Measuring scales	Weight (kg)	BMI = weight (kg) / height (m) ^2^
	Stadiometer	Height (m)	
	Tape measure	Waist circumference (cm)	Measurement instructions in Figure 4
Hypertension	Sphygmomanometer	Blood pressure (mmHg)	
Infection screening	HBsAg, Test It, Rapid, Turklabs. Turkey	Hepatitis B virus (HBsAg)	Fingerprick blood tests.
	Rapid Anti-HCV Test, InTec Products Inc. China	Hepatitis C virus (anti-HCV antibody)	
	INSTI Multiplex, Biolytical Laboratories, Canada	HIV and syphilis (anti-HIV-1, -2 and syphilis antibodies)	
Liver disease screening	Fibroscan™ (Echosens, France)	CAP (dB/m)	Steatosis
		VCTE (kPa)	Stiffness

Abbreviations: BMI - body mass index, CAP - controlled attenuation parameter, HBsAg - hepatitis B surface antigen, HCV - hepatitis C virus, HIV - human immunodeficiency virus, VCTE - vibration controlled transient elastography.

Venous blood tests will then be taken by a trained member of the study team. Combinations of blood tests will depend on the results of anthropometric data, fingerprick POCTs and Fibroscan™ results ( Table 1, Table 2).

**Table 2.** Table showing blood tests required for each participant group in the Hep-MoLo project.

Laboratory	Who to test?	Test	Comments
Clinical	Everyone	HbA1c	Mongolians may be at increased risk of diabetes ^36, 48^. Do not test those already diagnosed with diabetes (questionnaire Q5: yes)
		anti-HBs	Both negative: HBV susceptible, consider vaccination.
		anti-HBc	
		Lipid profile	Only test lipid profile if: BMI >23, Age >40, CAP >230 dB/m, Reactive HBsAg point-of-care test. Don’t lipid profile test if taking a statin (answer yes to questionnaire Q10)
	Reactive HBV POCT	HBsAg	Confirmatory testing.
		HBV viral load	assess stage of infection
		HBeAg/Ab	
		anti-HDV	HDV coinfection
		LFTs	Assess liver inflammation
	Reactive HCV POCT	anti-HCV	Confirmatory testing.
		HCV viral load	Assess for active infection.
		LFTs	Assess liver inflammation
	Reactive HIV POCT	HIV Ab/Ag test	Confirmatory testing.
		HIV viral load	
	Reactive syphilis POCT	Syphilis serology	Confirmatory testing and stage of infection.
	VCTE ≥10kPa	FBC	Further chronic liver disease assessment to assist hepatology referral.
		LFTs	
Research	Participant gives consent for research bloods	Serum	Lipidomics, viral sequencing, cytokine assays
		Whole blood	HLA sequencing
		PAXgene	Blood transcriptomics

*Abbreviations: anti-HBc - hepatitis B core antibody, anti-HCV - hepatitis C virus antibody, anti-HBe - hepatitis B e antibody, anti-HBs - hepatitis B surface antibody, anti-HDV - hepatitis D virus antibody, BMI - body mass index, CAP - controlled attenuation parameter, HbA1c - glycosylated haemoglobin, HBeAg - hepatitis B e-Antigen, HBsAg - hepatitis B surface antigen, HBV - hepatitis B virus, HIV Ab/Ag test - human immunodeficiency virus antibody/ antigen test, LFTs - liver function tests (alanine transaminase (ALT), alkaline phosphatase (ALP), bilirubin, aspartate aminotransferase (AST), gamma-glutamyl transferase (GGT)).*

Blood tests will be transported to a local clinically validated diagnostic laboratory and processed according to local protocols.

**6.2.2 Clinical follow-up**

A list of potential new conditions that may be diagnosed in this screening event are included in Table 3. Hep-MoLo team members will counsel participants appropriately, along with patient information leaflets on lifestyle advice, MASLD and viral hepatitis (available in English and Mongolian). Medically literate Mongolian speakers will be present at the event to translate as required.

**Table 3.** Follow-up guide for same-day screening tests.

Result	Follow up
Reactive POCT (HBV, HCV, HIV or syphilis)	• Inform the participant they have a “reactive” test, which needs to be confirmed with a venous blood test. • Give an information sheet and counsel participant. • Ensure we have the correct contact details so we can contact them with follow up information.
Possible metabolic dysfunction- associated steatotic liver disease (MASLD: CAP > 248 dB/m ^1^ + ≥ 1 CMF)	• Inform participant they have MASLD: signs of excess fat in their liver and a cardiometabolic risk factor (hypertension, overweight/obesity, diabetes or deranged lipids). • Give participant a MASLD/ cardiometabolic risk factor information leaflet ^46^. • **If VCTE <8kPa**, lifestyle and diet advice, with GP follow-up for management of cardiometabolic risk factors. • **If VCTE >8kPa,** refer to hepatology.
Possible advanced liver fibrosis or cirrhosis (VCTE > 10kPa)	• Inform participant they have signs of liver “stiffness” or “scarring”. • Inform participant we will need to refer them for specialist liver assessment. • Document on UCLH EPR: medication, alcohol and family history of liver disease.
Hypertension	In line with NICE hypertension guidelines ^49^ For BP measurement between 140/90 – 179/119mmHg: • Record the BP in both arms. If the difference is >15mmHg, take a second measurement. If the second is substantially different, take a third reading. Record the lower of the last two measurements. • Lifestyle advice: stop smoking, dietary advice (including reduced sodium, caffeine intake), exercise, weight management, alcohol. • Give participant patient leaflets and result and advice sheet ^50, 51^. • Verbal safety netting advice: chest pain, confusion, headache, difficulty breathing and/or rapidly swollen ankles - go to A+E • Advise participant to make appointment with GP to repeat BP and consider ongoing management plan. • Include in the post-study letter to the GP. For BP measurement of ≥180/120mmHg: • Repeat BP after 5–10 minutes, if the second measurement is substantially different than the first, measure for a third time and record the lower of the last two measurements. • Clinical review (doctor) – same day referral to A+E if: ∘ New onset confusion, pain, signs of heart failure or, ∘ Suspected phaeochromocytoma (e.g. postural hypotension, headache, palpitations, pallor, abdominal pain, diaphoresis). • Urgent email/ letter to GP
Overweight/ Obesity	• Inform participant: According to Asian ethnicity cut-offs, If BMI: ∘ 23–27.9 kg/m ^2^ - overweight ∘ ≥ 28 kg/m ^2^ - obese ^52^ • Increased risk of cardiometabolic complications if WC >80cm (women), >90cm (men) ^47^ • Give lifestyle advice and information leaflet – diet, exercise, alcohol.

*Abbreviations: A+E: Accident and Emergency services, BP - blood pressure, CAP - controlled attenuation parameter, CMF - cardiometabolic risk factor, GP - general practitioner, HDL - high density lipoprotein, LFT - liver function tests, MASLD - metabolic dysfunction-associated steatotic liver disease, NICE - national institute for health and clinical excellence, TG - triglycerides, VCTE - vibration controlled transient elastography, WC - waist circumference*.

Venous bloods will be reviewed by a study team member after the screening event. Possible blood test results and follow up actions are detailed in Table 4. New diagnoses of viral hepatitis, HIV, syphilis and diabetes will be discussed over the phone. For other results, participants and their GP will be informed of the results by letter, unless deemed by a clinician to require more urgent review. Explicit consent will be sought before informing the GP of viral hepatitis, HIV and syphilis results, in accordance with the Venereal Disease Act 1916.

**Table 4.** Interpretation and follow-up for venous blood results.

Venous Blood Result	Interpretation	Follow up
Reactive POCT, but negative confirmatory serology	False positive POCT	Inform the participant via phone.
Reactive HCV POCT, anti-HCV serology positive, HCV viral load/PCR negative	Past HCV infection	Inform the participant via phone. Ask if they know about diagnosis or have received treatment in the past. No need for referral if normal liver profile and Fibroscan™.
Reactive HCV POCT, anti-HCV serology positive, HCV viral load/PCR negative	Current HCV infection	Inform participant via phone Ask if they know about diagnosis or have received treatment in the past. Explain this is now a treatable condition with 8–12 weeks of tablets. Explain that we will refer them to Mortimer Market Centre for at least the initial workup, unless they have a strong preference to be seen elsewhere. Following this they can be referred to a more convenient location if they prefer.
Reactive HBV POCT, HBsAg positive and/or HBV viral load detected	Current HBV infection	Inform the participant via phone. Ascertain if this is a new diagnosis, or they know about diagnosis but are not currently under follow up. Referral as per current HCV infection.
Negative HBV POCT, Anti-HBc negative, Anti-HBs negative	Susceptible to HBV infection	If there is no history of HBV vaccination, inform the participant they are susceptible to HBV infection. If they are at risk e.g. sexual or household contacts, then they should be vaccinated (in which case this may be given by GP or viral hepatitis clinic of contact).
Negative HBV POCT, Anti-HBc positive, Anti-HBs negative	Previous HBV infection	Explain they have been exposed to HBV in the past but do not have a current “active” infection. In the future if their immune system is suppressed, they will need consideration of treatment to prevent reactivation. They do not need vaccination.
Negative HBV POCT, Anti-HBc negative, Anti-HBs positive	Previous HBV vaccination	Check if they have received HBV vaccination. They have immunity to HBV.
EIA/CMIA/TPPA positive, RPR negative	Past or early syphilis	Participants can self-refer to local sexual health clinic (or we can refer). Give information about local sexual health clinics.
EIA/CMIA/TPPA positive, RPR positive	Current syphilis infection	
POCT positive, serology tests negative	False positive POCT result	Explain false positive result to participant. No need for referral.
Deranged lipid profile – any result out of normal range	Dyslipideamia (specify which type e.g. hypercholesteraemia)	Write to GP and participant with results. Highlight to GP for possible specialist referral if: - Triglycerides >10mmol/l - Non-high density lipoprotein cholesterol >7.5mmol/L or total cholesterol > 9mmol/l Refer directly for urgent specialist review if: - Triglycerides >20mmol/l in absence of poor glycaemic control or excessive alcohol intake.
HbA1c 42–47 mmol/mol	Prediabetes	- Inform the participant via phone that they are at higher risk of developing diabetes. - Email or send participant information leaflet ^53^. - Lifestyle advice – diet, weight management. - Inform GP via letter.
HbA1c 48 mmol/mol or higher	Suspected diabetes (confirmed if symptoms present)	- Inform the participant via phone. - Check if they have any recent blood transfusion, long term corticosteroid use, haemoglobinopathy (e.g. thalassaemia), iron or B12 deficiency, splenectomy, renal dialysis, rheumatoid arthritis which may affect result. - Are they known to have diabetes or on medication for diabetes? - Ask about symptoms (polyuria, polydipsia, peripheral neuropathy) - Advise the participant to make an appointment with the GP for assessment, may consider diet/ lifestyle advice only or may start medication. - Email participant patient information leaflet ^54^. - Inform GP via letter (email or phone if symptoms which may require a more urgent review).
HbA1c >120mmol/mol	Suspected diabetes (very high)	Urgent clinical review: check for symptoms over the phone and consider either urgent GP appointment or ambulatory care appointment for urine ketones and treatment ^55^.
Deranged liver profile	Depends on individual result	Will need consideration on a case-by-case basis by the clinician. The participant will be contacted for a phone assessment, then an appropriate follow up plan arranged.

*Abbreviations: Anti-Hbc - hepatitis B virus core antibody, Anti-HBs - hepatitis B virus surface antibody, anti-HCV - hepatitis C antibody, CMIA - chemoluminescent micropartical assay, EIA - enzyme immunoassay, GP - general practitioner, HbA1c - glycosylated haemoglobin (haemoglobin A1c), HBsAg - hepatitis B surface antigen, HBV - hepatitis B virus, POCT - point-of-care test, RPR - rapid plasma reagin*.

**6.2.2 Research Follow-up**

We will contact the specialist care providers for participants diagnosed with HIV, HBV, HCV or HDV to ascertain linkage-to-care within 3 months, and retention-in-care at 12 months post diagnosis. The Find & Treat team will assist with loss to follow up, if required.

Anonymised data will be assimilated and shared (including in publication format) to support research insights, and clinical and public health service development. We will retain anonymised data and samples for 10 years such that material can be reused for future analyses relevant to chronic BBV infection and liver disease, subject to terms of consent and ethical permission (those who have consented for research).

*6.3 Laboratory research domain (part 2)*

Participants who indicate their consent to take part in laboratory research will have three additional blood tubes collected ( Table 2):

- Serum for pathogen sequencing, metabolomics, immune biomarkers, viral biomarkers (e.g. HBV core-related antigen, HBcrAg).
- Whole blood for host HLA sequencing.
- PAXgene™ blood RNA tube (BD biosciences Cat No. 762165) for transcriptomics - everyone with a positive viral hepatitis POCT, ~ 25 people with a negative POCT, ~ 25 MASLD, ~ 25 no MASLD/HBV. Numbers are limited due to the cost. These numbers will be reviewed and may be adjusted according to recruitment.

Laboratory research bloods will be processed and analysed at the Francis Crick Institute in a designated and approved containment level 3 (CL3) facility. Serum will be processed and stored on the same day at -80°C. Whole blood and PAX gene tubes will be stored at -80°C on the day of collection until processing. Samples will only leave the CL3 environment when they have been treated to inactivate infectious material based on existing laboratory protocols which have been approved by internal Crick Health and Safety and by external review.

Research analysis will be undertaken including:

- **HBV sequencing:** we will aim to generate full genome HBV sequences from viraemic samples, using an optimised pipeline (‘HEP-TILE’) which combines PCR amplification, and long-read (e.g. Nanopore) sequencing ^56, 57^. We will store samples for possible future sequencing of other viruses (e.g. HIV, HDV, HCV).
- **Lipidomic analysis:** In collaboration with the Metabolomics Scientific Technology Platform at the Crick, lipids will be extracted from serum samples using a modified Bligh-Dyer method and analysed via Liquid Chromatography-Mass Spectrometry ^58^. We will determine the relationship between lipid peripheral profiles and virologic outcomes, liver fibrosis/steatosis.
- **Measurement of a panel of host and viral biomarkers** pertaining to the development of liver disease (see extended data for further assays planned depending on preliminary results and funding ^46^)
  ∘ **Viral antigens** (quantification of HBV antigens including HBsAg, HBeAg, HBcrAg).
  ∘ **Viral nucleic acids** (quantification and/or sequencing of viral DNA / RNA for any hepatitis viruses detected in the sample).
- **Ascertainment of host genetic background** focusing on the MHC Class I region (HLA genes) which are known to be important determinants of immune response to vaccination and infection ^25, 59^, but also generating information on the whole genome to undertake lineage correction.

For assays that are not available in-house at the Francis Crick Institute, we will send samples to collaborating laboratories for additional tests (in the UK or internationally) such as quantification of HBcrAg. Any samples sent to other laboratories will be under the terms of a Material Transfer Agreement (MTA), and participant information / consent will include their permission for samples to be shipped if required. No identifying data will accompany samples shipped for research purposes.

*6.4 Qualitative domain (part 3)*

During the screening event, participants will be asked if they consent to future contact to be invited into a qualitative study ^60^. We will use methods similar to those previously published, in a study which investigated barriers and opportunities to HBV testing in a UK Somali population ^61^.

We will conduct 2–4 focus groups of 6–15 participants (number of groups will be limited by thematic saturation, willing participants, time and cost) ^62^. We will use purposive sampling to identify a range of community members to achieve a sample representative of population demographics to assimilate views on chronic viral hepatitis testing, access to healthcare and community health priorities. We will ask participants their experiences and attitudes towards community healthcare screening. To avoid the risk of accidental disclosure of infection status, we will only invite participants who are not living with a BBV to take part in focus groups.

We will also perform a semi-structured 1:1 interview of a sample (~5) people diagnosed with chronic viral hepatitis to explore barriers and facilitators to diagnosis, linkage-to and retention-in-care, stigma and discrimination, and other topics relevant to chronic viral hepatitis diagnosis and management.

Semi-structured topic guides will provide a starting point for focus groups and interviews ^46^. One-to-one interviews will be conducted in a private and confidential environment, or via phone, depending on the availability and preference of the participants and facilitators. In-person focus groups will be held at a convenient London location. Where possible, interviews and focus groups will be facilitated or assisted by trained members of the Mongolian community. We will remunerate community facilitators in line with NIHR ‘Involve’ guidance ^63^. Participants will be made aware of confidentiality in the participant information sheet and consent form and reminded of confidentiality required in the focus groups.

We will do an online debrief for facilitators after each screening event to understand what went well and what could be improved for the next event. Verbal consent will be taken to record this on Microsoft Teams™. We will also invite feedback via email for anyone who can’t attend the debrief meeting.

### 7. Sample size

Our sample size will be pragmatically determined by the size of the community who engages with the research. Based on an estimated Mongolian community of 1300 people living in London [personal communication, Mongolian Embassy], we estimate 70% of these to be adults aged 18+ (n=910), and of these we will aim to recruit and engage 500 people.

**Figure 5.**
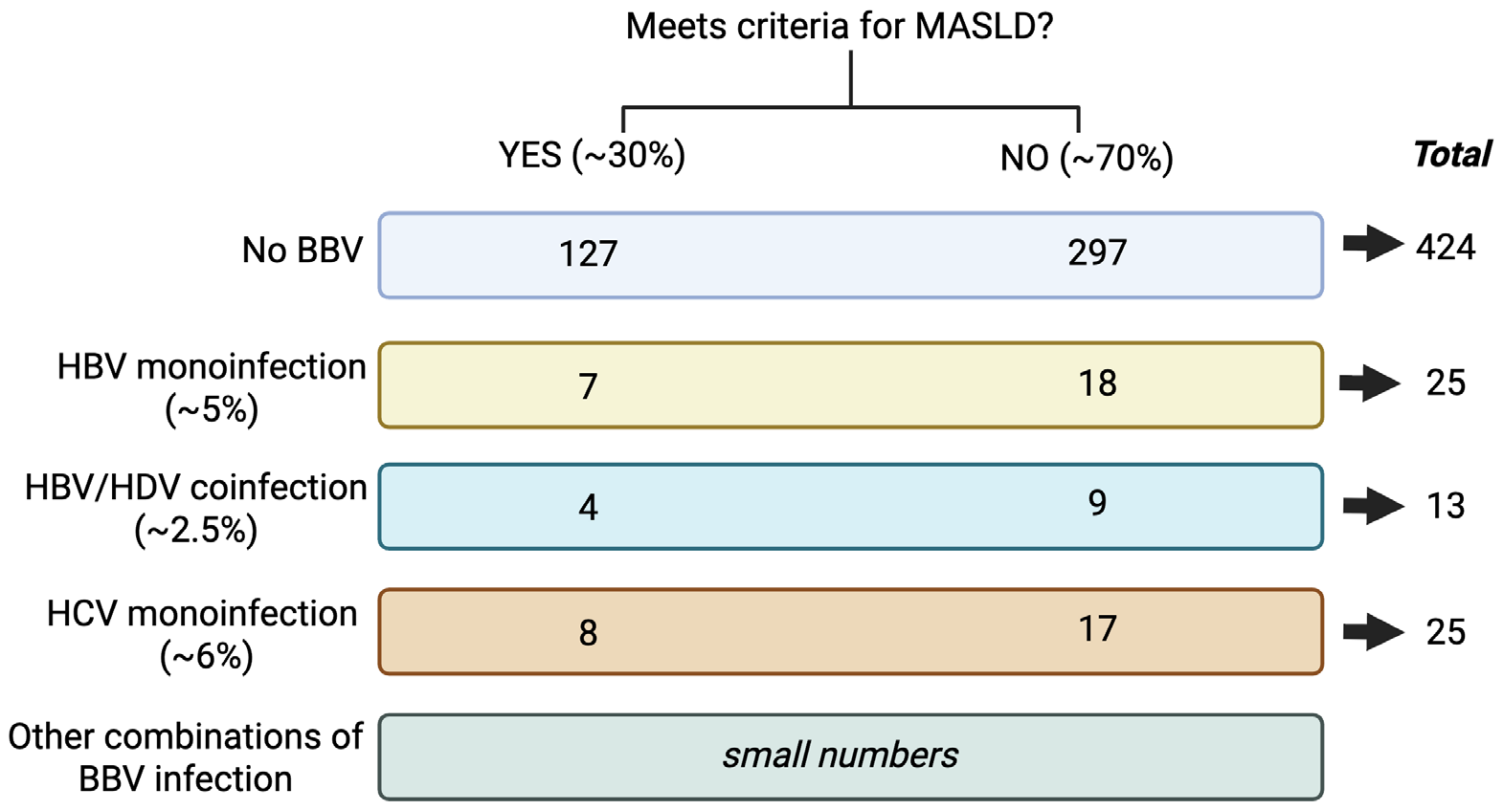
Schematic illustrating analysis groups and expected numbers in each category to be recruited through the Hep-MoLo study. Estimated numbers in each group are based on published data and a sample size of 500 ^30– 33, 35, 65– 68^. Other combinations include HBV/HCV, and human immunodeficiency virus (HIV) monoinfection or coinfections. Created with BioRender.com. Abbreviations: BBV - blood borne virus, HBV - hepatitis B virus, HCV - hepatitis C virus, HDV - hepatitis D virus, MASLD - metabolic dysfunction-associated steatotic liver disease.

Approximate estimated numbers per recruitment group based on published prevalence estimates with an expected screening number of ~500 can be reviewed in Figure 5 and under “Supplementary Tables” in the extended data ^46^.

This is a small study and therefore will have limited statistical power. We will aim to calculate prevalence of HBV and MASLD within the London Mongolian community, while noting the limitation of a selection bias among people who self-select to attend screening events.

Using the formula for calculating sample size for prevalence studies n = Z^2P(1-P)/d^2, where:

- n: is the sample size
- Z: is the statistic for the confidence level (1.96 i.e. 95% confidence interval)
- P: is the expected prevalence (7.5%)
- d: is the precision (0.05, +/- 2.5%)

Using the above estimated parameters, a sample size of approximately 427 is needed to determine a prevalence of HBV of 7.5% with 95% confidence. A smaller sample size (323) is needed to calculate the prevalence of MASLD, since it is a more common condition. The rest of the analysis of the clinical parameters will be descriptive i.e. demographics, comorbidities, linkage-to and retention-in-care for people diagnosed with chronic viral hepatitis, therefore no specific sample size or power calculation is required. There is a precedent for similar observational, descriptive studies including chronic viral hepatitis screening studies in the Mongolian community in the U.S., Spain and Sweden and migrant populations in Spain (HBV-COMSAVA study) ^30, 31, 33, 64^.

For this pilot study, laboratory analyses will be exploratory, investigating trends using correlation with lipidomic, transcriptomic and sequencing data which can be used to power future studies, such that we provide foundations for a more targeted approach. A particular benefit of this study is that blood samples from control groups without liver disease are from the same population, processed in identical conditions, with the same collected metadata as the liver disease groups. Appropriate controls are rare and difficult to obtain in laboratory-based studies using human samples.

### 8. Data management, sample use and storage

The study is compliant with the requirements of General Data Protection Regulation (GDPR) (2016/679), the UK Data Protection Act (2018) and Human Tissue Act (2004). All investigators and study site staff will comply with the GDPR requirements with regards to the collection, storage, processing and disclosure of personal information, and will uphold the Act’s core principles. UCL is the data controller and data processor. Personal data collected in this study is detailed in Table 5.

**Table 5.** Personal data fields to be collected and recorded within the Hep-MoLo project.

Data Source	Data field	Special Category *
Clinical data (screening event)	Age	N
	Gender	N
	Sexual orientation	Y
	Country of birth	N
	Years of first arrival in the UK	N
	Co-morbidities	Y
	Current medication	Y
	Alcohol intake	Y
	Smoking status	Y
	Dietary information	Y
	Hepatitis B vaccination status	Y
	Any relatives living with chronic viral hepatitis	Y
	Information about medical procedures which may increase viral hepatitis risk e.g. dialysis, obstetric care, operations, blood transfusions outside the UK	Y
	Weight, height, BMI, waist circumference	Y
	Liver stiffness	Y
	Controlled attenuation parameter	Y
	Viral hepatitis status (HBV/HCV/HDV)	Y
	Associated viral parameters if positive viral hepatitis result (HBV viral load, HBsAg, HBeAg, HBcAb, HCV viral load)	Y
	HIV status	Y
	Syphilis screening result	Y
	Liver function tests	Y
	HBa1c	Y
	Lipid profile	Y
Laboratory research data	HBV genomic sequencing data	Y
	Lipidomic profile	Y
	Cytokine profile	Y
	HLA sequencing data	Y
	Whole blood transcriptomic data	Y
Qualitative data	Audio recordings of focus groups and interviews	Y

* *Data defined by UK General Data Protection Regulation as likely to be more sensitive and therefore requiring extra protection* ^69^. *Abbreviations: BMI - body mass index, HbA1c - haemoglobin A1c (glycosylated haemoglobin), HBsAg - hepatitis B surface antigen, HBeAg - hepatitis B e antigen, HBcAb - hepatitis B core antibody, HIV - human immunodeficiency virus, HLA - human leukocyte antigen, N - no, Y – yes.*

The Chief Investigator will ensure there are adequate quality and number of monitoring activities conducted by the study team. This will include adherence to the protocol, procedures for consenting and ensuring adequate data quality. The Chief Investigator will inform the sponsor if any concerns arise.

Sources and types of study data are summarised in Table 5. Direct access to study data will be restricted to authorised members of the study team to enable them to carry out their duties, including clinical follow up, and case report forms (CRF) entry. For data analysis, pseudonymised identifiers will be used. Linkage back to identifiable source data will only be possible through authorised team members who hold clinical contracts. Access will also be granted to authorised representatives from the Sponsor, host institution and regulatory authorities to permit study-related monitoring, audits and inspections. The Chief Investigator will be the Data Custodian of the study.

**Figure 6.**
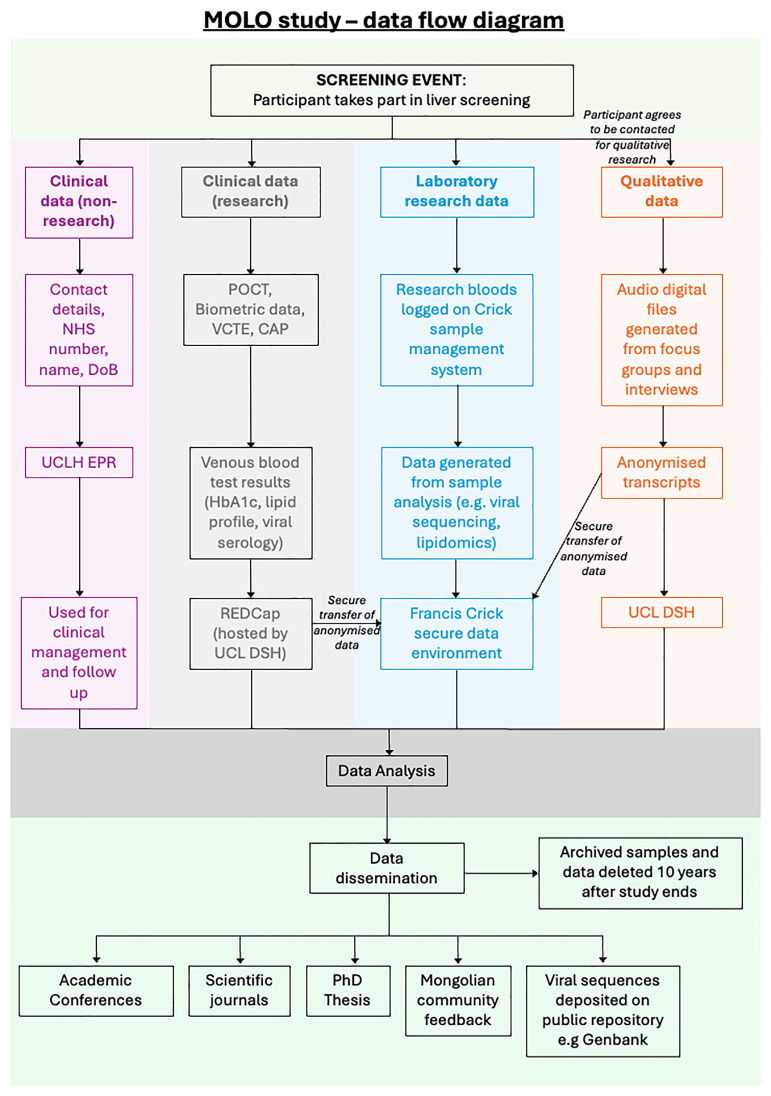
Schematic illustrating data flow within the Hep-MoLo project. *Abbreviations: CAP - controlled attenuation parameter, DoB - date of birth, EPR - electronic patient record, HbA1c - haemoglobin A1c (glycosylated haemoglobin), NHS - National Health Service, UCL DSH - University College London Data Safe Haven, VCTE - vibration controlled elastography*.

Figure 6 describes data flow. At study enrollment the participant will be issued a participant identification number. The participant ID will follow the format MOLO-X-YYY, where X is screening event number (1–5) and YYY is participant number (1–500). We will hold multiple screening events but individual participants will only be screened once. The participant’s informed consent form (ICF) will carry their name, initials for each item consented, signature and date. The electronic ICF will be via UCL Data Safe Haven (DSH), but printed copies will also be available in case of technical issues or patient preference. Printed ICFs will be uploaded to the participant's UCLH electronic patient record (EPR).

- ***Clinical data***: The research team will fill electronic CRF using a secure web application for building and managing online surveys and databases (Research Electronic Data Capture ‘REDCap’) with restricted access and password protection, hosted by UCL Data Safe Haven ^70^. Some data will be entered on the screening day, and some will need to be populated once blood results are available on the UCLH EPR. Linkage-to and retention in care data will be recorded a year after the screening event following phone calls to the viral hepatitis clinics to ascertain clinic attendance. Data will be stored on UCL Data Safe Haven, which has been certified to the ISO27001 information security standard and conforms to NHS Digital’s Information Governance Toolkit. ICFs and electronic CRFs will be stored on REDcap, held within UCL Data Safe Haven. Any email electronic transfer of data will be with secure NHSmail email addresses.
- ***Research laboratory data:*** All study specific research samples will be labelled with study number, date of collection, and participant initials. Upon arrival, samples will be logged in the sample management system. Data generated from laboratory research will be stored in the secure data environment hosted by the Francis Crick Institute. Pseudonymised clinical meta-data will be transferred from UCL to the Francis Crick Institute for analysis.
- ***Qualitative data:*** Approximately 8 hours of audio files in an appropriate format (e.g. mp3) will be stored on UCL Data Safe Haven. Participants will be asked not to share personal identifiable information during audio recording (e.g. full name, specific locations or addresses), however if personal information is recorded, this will be redacted from recordings before transcription. Anonymised transcripts will be created for analysis.

Clinical samples will be transported, processed, stored and destroyed according to UCLH laboratory SOPs. Research samples will be stored at the Francis Crick Institute for up to 10 years. They will be de-identified and labelled with a pseudonymised code. Samples may be shared with collaborators according to MTA, in line with ethical approvals and informed consent provided by study participants.

### 10. Data analysis/statistical plan

We will classify participants according to their BBV and MASLD status ( Figure 5). We will focus on analysis of PLWHB +/- MASLD, people with MASLD only, and people without MASLD or HBV, as we expect most participants to fall into these categories.

*10.1 Clinical data analysis*

We will describe:

- Prevalence of HBV, HBV+HDV, HCV infection and MASLD. Appropriate weighting strategies will be applied and 95% confidence intervals calculated.
- Population demographics.
- Liver disease markers (elastography and CAP scores, blood liver profile).
- Linkage-to-care (3 months) and retention-in-care (12 months) for people diagnosed with viral hepatitis.

We will compare liver disease severity and viral parameters between PLWHB +/- MASLD, using control groups (MASLD only, no HBV or MASLD) to explore the impact of MASLD on chronic viral hepatitis. Depending on participant recruitment numbers and demographics, we will attempt to match the control to the HBV groups 1:1 for age/sex. Statistical tests will include Fisher’s Exact Test / Chi-Squared for univariate analysis of binary variables, Mann-Whitney test for quantitative variables.

*10.2 Laboratory data analysis*

We will compare serum lipidomic profile and cytokine analysis to explore key differences between PLWHB +/- MASLD, with MASLD only and no HBV/MASLD age- and sex-match control groups using multi-dimensional statistical modelling.

We will sequence HBV (in viraemic participants) and investigate for associations with liver disease severity, MASLD and response to antiviral treatment. HBV sequencing data analysis will be in collaboration with the biostatistics and genomics science technology platforms at the Crick, using a pipeline developed in collaboration with the ARTIC team at the University of Birmingham, UK (with open-source code) ^71^. We will also sequence host HLA class I genes, and investigate for associations with disease stage, viral markers and response to antiviral treatment. We recognise that we are unlikely to have power to interpret sequencing data in this small clinical cohort, however, they will contribute to similar data from larger clinical cohorts.

We will analyse laboratory and clinical data using R studio, Prism GraphPad and relevant software suitable for multiparametric analysis ^72, 73^. Anonymised demographic, clinical and laboratory data may be pooled with data from other studies to facilitate cross-comparison between populations in different settings, and/or to expand datasets and improve statistical power for the identification of relevant biological signals.

*10.3 Qualitative data analysis*

We will analyse qualitative data using thematic framework analysis techniques ^74, 75^. Transcription will be performed by either a research team member or a professional service, depending on human resource availability and coding and thematic analysis will be performed by two members of the research team. Anonymised audio recordings and written accounts/field notes will be transcribed into Word documents, with transcription, coding and analysis being facilitated by appropriate software (e.g. NVivo).

### 11. Ethical considerations

*11.1 Confidentiality*

Our PPI survey highlighted the importance of privacy during the screening event, and we recognise this may be a challenge within a small community and in an outreach setting. To ensure confidentiality is upheld, we have chosen a venue with multiple rooms available to allow private consultation and identified medical Mongolian speakers from outside of the immediate London community to help facilitate the screening events. We will need to contact the participant’s GP in order to update them with results and to ask for follow-up of newly diagnosed conditions (e.g. hypertension or hypercholesterolaemia). We will obtain participant consent before contacting their primary care provider.

*11.2 Point of care versus venepuncture testing*

We opted to use POCT with confirmatory venous serology for positive results only for the following reasons:

- Community preference:
  ∘ Mongolian members of our study team and feedback from the community survey expressed that the community would prefer as much information as possible on the same day.
  ∘ Previous learning from similar community screening projects informed POCT e.g. COMSAVA hepatitis screening, Spain and Chagas Hub, London ^64, 76^.
- Decentralisation:
  ∘ Use of POCT supports decentralisation of care to make diagnosis and linkage to care more accessible.
- Clinical and research stratification:
  ∘ Knowing the diagnosis on the day can help us focus on ensuring the correct information is given to participants with a new diagnosis, taking the appropriate confirmatory bloods and making timely referrals to other services.
  ∘ Real-time stratification will allow us to collect research bloods for all those with BBVs but limit the number of non-BBV controls, thus using resources more effectively and not collecting unnecessary samples.
- Favourable test performance characteristics:
  ∘ Review of the POCT accuracy studies in the context of expected prevalence in the Mongolian community (HBV 10%, HIV <0.1%, HCV 6%) and 500 participants found low risk of false positive and negative results ( Table 6).
  ∘ We will counsel participants about the possibility of false positive results and confirmatory tests will be sent such that all results will be validated in a clinical diagnostic laboratory facility.

**Table 6.** Performance of point of care tests being used in the Hep-MoLo study.

Test	Expected BBV prevalence	Sensitivity	Specificity	False positive number *	False negative number *
**Test-it rapid HBsAg** ^a^ (Turklabs, Turkey)	10%	98.3%	99.5%	2	1
**INSTI Multiplex HIV/Syphilis test** ^b^ (Biolytical Laboratories, Canada)	<0.1%	98.2%	100%	0	0
**Rapid Anti-HCV Test** ^c^ (InTec Products Inc, China)	6%	100%	99.7%	2	0

* number of people rounded to the nearest whole person. ^a^calculations based on a comparative study of rapid tests on serum and plasma samples collected in France and Cameroon ^77^. ^b^Field evaluation of rapid test on samples collected using fingerstick whole blood in U.S clinics. ^c^World Health Organization product report of test, which is on the pre-qualified list of *in vitro* diagnostics ^78^. Abbreviations: BBV - Blood Borne Virus, HBsAg - hepatitis B surface antigen, HIV - human immunodeficiency virus, anti-HCV - hepatitis C virus antibodies.

*11.3 Participant distress*

There is a possibility that a participant may experience distress or anxiety. Due to the POCT approach, participants may receive a new diagnosis on the day of the screening, which may cause distress at the time or subsequently. Our experienced outreach team members are skilled in giving new diagnoses and supporting participants through follow up. We have a distress protocol to manage a situation where a participant becomes distressed during the study which is available to view online ^46^.

*11.4 Participant withdrawal*

Participants who engage with clinical testing will be linked to services and provided with information, support and encouragement to engage in relevant clinical follow-up.

For those who provide consent for research, they will be informed that they can withdraw from the research component of the study at any time, and this will not affect the standard of clinical care they receive. If the participant chooses to withdraw from the study, they will have two options:

1. Withdraw from the study and not be approached for further information. However, the samples and data already obtained, including audio recordings from focus groups may still be retained and used.
2. Request no further contact and for their samples and data to be destroyed. Data and samples will be destroyed if they have not already been used / published as part of an analysis.

### 12. Plans for dissemination of results

Results will be disseminated at clinical and academic meetings, conferences and published in scientific journals following peer-review. Where possible, results will be shared on pre-print servers ahead of peer review (e.g. MedRxiv, BioRxiv). Reports and materials that are not peer reviewed will be made available for reuse by sharing in the public domain (e.g. using Figshare). We will improve visibility of results by working with the comms teams of our institutes and sharing on professional networks (e.g. LinkedIn). We will also share the results with community members and participants via social media, and the MCO e.g. in the form of a community information event.

## Discussion

This protocol describes a project to hold a series of liver health screening events among the London Mongolian community, accompanied by laboratory and qualitative research components.

Hep-MoLo benefits from broad experience within a multidisciplinary research team and is strengthened by community co-design, and by a mixed methods approach, which enables implementation of a pilot public health intervention while also creating a platform for better understanding of lived experience to enhance access to healthcare. From a laboratory perspective, a well characterised cohort of people with and without liver disease enables clear experimental groups and a rare well-matched control group from the same population.

This project supports England’s progress towards the WHO targets to eliminate viral hepatitis as a public health problem by 2030, specifically a 90% reduction in incidence and 65% reduction in mortality from hepatitis B and C compared to 2015 ^79^. While significant progress has been made in England for HCV ^10^, HBV remains off target to reach SDG30 goals. Hep-MoLo will pilot methods of improving progress towards elimination goals, suggested by the British Liver Trust, for example, raising awareness of HBV among at-risk communities, increasing community outreach and availability of HBV testing in different settings ^80^.

Prevalence estimates for viral hepatitis and MASLD, and generalisability of results may be impacted by selection bias, since individuals who self-select to attend a liver screening event are unlikely to be representative of the whole population. The laboratory component of Hep-MoLo may also be limited by sample size, since the Mongolian community is relatively small and it is difficult to predict community uptake. However, we may be able to combine analysis with data from similar clinical cohorts to increase power and therefore meaningful clinical conclusions.

Hep-MoLo is affected by several limitations common to many liver disease studies, for example reliable measurement of important variables which impact liver disease, such as duration of viral hepatitis infection or MASLD and accurate alcohol intake estimation, and cross-sectional study design, which limits conclusions about causality.

## Conclusion

We have described a mixed-methods protocol for community outreach-based viral hepatitis screening among Mongolians living in London. Our approach enables multi-disciplinary collaborations and provides a strong basis for ongoing public-academic-clinical partnerships.

## Data Availability

This protocol contains no primary data. Information collected through public engagement prior to the project is available at https://doi.org/10.6084/m9.figshare.26312389 ^38^; no further metadata are publicly available due to confidentiality. New data generated by the project are not available at the time of protocol publication but will be released as supporting metadata in future publications, in accordance with REC approval and FAIR data principles, with data archived in secure institutional repositories. Where full datasets cannot be made available, this will be explained and contact details for the chief investigator will be provided for enquiries. Extended data (study documents) are available to view online ( https://doi.org/10.6084/m9.figshare.30328327) ^46^. No other datasets are used in the creation of this protocol.
