# Supplementary material for: Viral hepatitis and liver disease in the Mongolian community in London ('MoLo'): A mixed methods study protocol to investigate the epidemiology and burden of disease to inform clinical and public health interventions
Source: Wellcome Open Res. 2026 Jan 23;11:72. [Version 1] doi: 10.12688/wellcomeopenres.25134.1 (PMC13305554; doi:10.12688/wellcomeopenres.25134.1)
Supplement: Peer review history

# Reviewer report: Reviewer response for version 1

**DOI:** 10.21956/wellcomeopenres.27707.r152850

**Published:** 2026-06-25

**Authors:** Omphile E Simani

**Affiliations:** 1 Sefako Makgatho Health Sciences University, Pretoria, South Africa

Referee Report

 This manuscript presents the Hep-MoLo study protocol, a community-based liver health screening programme targeting the Mongolian population living in London. The protocol aims to estimate the prevalence of viral hepatitis, including HBV, HCV and HDV, and metabolic dysfunction-associated steatotic liver disease (MASLD), while also evaluating the implementation of a culturally tailored screening and linkage-to-care model. In addition, the study incorporates exploratory laboratory and qualitative components intended to provide further insight into biological, clinical and community-level factors associated with liver disease in this population.

 Overall, the protocol is well structured, clinically relevant and operationally detailed. A clear strength of the study is the community-engagement approach, which is likely to improve acceptability, participation and linkage to care among a population that may experience barriers to mainstream healthcare services. The clinical pathway, ethical considerations, participant management procedures and implementation processes are generally described with sufficient clarity.

 The rationale for the study is clearly presented and addresses an important public health gap in an underserved migrant community. The objectives are broadly appropriate and are well aligned with the screening and implementation components of the study. However, the link between the primary public health objectives and the more extensive laboratory-based investigations requires clearer justification. In particular, the authors should explain more explicitly how the biomarker, sequencing, immunological and other exploratory analyses will contribute to the overall purpose of the protocol and how these findings may inform future research or intervention development.

 The study is described as a mixed-methods cross-sectional study. This design is appropriate for estimating disease prevalence, identifying screening and care gaps, and assessing implementation outcomes within a community-based programme. However, the study appears to function primarily as a screening and implementation study with embedded qualitative and laboratory components. Therefore, while the design is suitable for the main public health questions, it is less appropriate for addressing causal questions relating to biological pathways, disease progression, or interactions between liver disease processes.

 The manuscript would be strengthened by clearly distinguishing the primary objectives from the exploratory objectives. For example, prevalence estimation, screening uptake, linkage to care and implementation outcomes should be presented as the core study aims, whereas biomarker discovery, sequencing and immunological analyses should be framed as exploratory and hypothesis-generating. This distinction is important because the current design does not support causal inference, and the laboratory components should not be interpreted beyond what the study design can reasonably support.

 In addition, the mixed-methods component requires further clarification. Although quantitative, qualitative and laboratory data are included, the protocol does not clearly describe how these different forms of evidence will be integrated. The authors should specify the mixed-methods framework being used and indicate whether integration will occur during data collection, analysis, interpretation or reporting. This would improve the coherence of the study and help readers understand how the different components will collectively inform liver health, healthcare access and barriers to care within the target population.

 The methods are generally described in sufficient detail to allow replication. The recruitment strategy, clinical assessments, laboratory testing procedures, qualitative data collection, ethical safeguards and participant referral pathways are clearly outlined. These details support the reproducibility of the screening and implementation aspects of the protocol.

 Nevertheless, several minor methodological clarifications would further strengthen the manuscript. These include additional detail on HDV RNA testing procedures, clinical management pathways for participants diagnosed with HDV infection, referral processes for participants diagnosed with syphilis, blood collection volumes, and the statistical approach for handling multiple comparisons in exploratory laboratory analyses.

 The protocol appropriately states that no datasets are currently available, as the study has not yet been conducted. However, the data-sharing statement could be made more informative and aligned with FAIR principles. The authors should specify what types of data will be shared, such as de-identified participant-level data, metadata, codebooks, statistical code or summary-level datasets. They should also clarify any restrictions related to participant confidentiality, ethical approvals, data governance or controlled-access arrangements.

 Additional comments

 A further limitation that should be discussed more explicitly is the potential for self-selection bias introduced by voluntary community-based screening. Individuals who choose to participate may differ from non-participants in health-seeking behaviour, perceived risk, awareness of liver disease or engagement with healthcare services. This has implications for the interpretation and generalizability of prevalence estimates. The manuscript would also benefit from a stronger explanation of how the study findings may be translated into public health action. For example, the authors could clarify how the results may inform screening policy, community awareness campaigns, linkage-to-care models or culturally tailored interventions for migrant communities.

 Conclusion

 In conclusion, this is a strong and well-developed protocol with clear clinical, public health and implementation relevance. The main issues relate to conceptual alignment between the objectives and study design, clearer framing of the exploratory laboratory analyses, specification of the mixed-methods integration approach, and more detailed information on data-sharing and translation of findings into practice. These concerns do not require major methodological changes, but they should be addressed through clearer justification, improved framing and more explicit reporting.

Is the study design appropriate for the research question?

Partly

Is the rationale for, and objectives of, the study clearly described?

Yes

Are sufficient details of the methods provided to allow replication by others?

Yes

Are the datasets clearly presented in a useable and accessible format?

Not applicable

Reviewer Expertise:

HIV and viral hepatitis infections, genomics, surveillance, diagnostics, and drug resistance.

I confirm that I have read this submission and believe that I have an appropriate level of expertise to confirm that it is of an acceptable scientific standard.

# Reviewer report: Reviewer response for version 1

**DOI:** 10.21956/wellcomeopenres.27707.r146789

**Published:** 2026-03-13

**Authors:** Anders Boyd

**Affiliations:** 1 Public Health Service Amsterdam, Amsterdam, The Netherlands

In this protocol manuscript, Martyn et al describe the procedures of what they term a mixed-methods study to examine facets of viral hepatitis and metabolic liver disease in the Mongolian community in London. The eventual goal is then to inform future interventions and care pathways specific to the Mongolian community. The protocol is very well described and easy to follow. There are a few broad issues that need some consideration.

 The mixed design usually involves some qualitative investigation that feeds into formulating a separate, quantitative investigation. Here, the links are somewhat reversed and populations for parts 2 and 3 are nested in the whole of part 1 (if we look at Figure 1). In my opinion, this is a community screening project that will leverage its recruitment to conduct nested studies on biological/laboratory and qualitative research. The framing could be changed.

 It is somewhat unclear how the information from these studies together will inform the various stakeholders. Higher prevalence of these diseases likely requires broader screening- and future interventions could be improved with the qualitative component. But what if the prevalence is very low? Does this mean that future interventions are unnecessary? Although this scenario is unlikely, it should be considered. Furthermore, what information will be used from the laboratory studies to inform future research/interventions?

 For the record: I completely agree with the authors on avoiding sample size calculations in case there are any doubts by others. The sample size calculations for these types of studies are challenging and mostly rely on convenience samples. I appreciate that the authors examined the numbers needed with respect to precision, yet I find it hard to understand what a clinically meaningful precision estimate really is.

 Minor comments:

 - The title has the acronym “MoLo”, but the study is called the “Hep-MoLo” in the rest of the manuscript.

 - The title could exclude: “To inform clinical … interventions” as this will only be a direct result of the study, not the study itself.

 - List of abbreviations: Add “SOP” and define this acronym in the text.

 - p6, para 1. Not sure you need to mention the reason for the change to MASLD.

 - p6, para 2. “inclusion health” populations is mentioned here but introduced later in the document. Could you please define here?

 - p6, para 3. Paragraph on evidence gaps could be outlined to match the study components.

 - p10, para 1. Suggest briefly stating that standard of care will not change if consent is refused.

 - Table 2. Some participant groups would like to know how much blood is being taken exactly. You could add this information here.

 - Table 2. No HDV RNA in the event of positive anti-HDV?

 - Table 4. For the group without immunity against HBV and no past infection- given the high prevalence of HBV infection in the community, should everyone be offered vaccination?

 - Table 4. No intervention for those with active HDV infection? There was mention of HDV treatment availability in the introduction.

 - p14, para 2. Is there linkage-to-care with syphilis?

 - p15, para 6. “… a prevalence of 7.5% with 95% confidence interval of±2.5%.”

 - p17, section 10.1. I imagine there will be multiple comparisons? Reviewers may bring up adjusting p-values for multiple comparisons. I would be upfront on whether you plan on doing that or not.

 - p17, section 10.1. It is also stated “HBV groups”. Is this meant to be HBV±MASLD groups?

 - Figure 6. Are there other governmental or public health agencies involved in the dissemination plan?

 - p20. There is discussion that these plans support public health England’s progress toward viral hepatitis elimination. Given the small population of Mongolians living in London- even if all the cases of HBV±HDV and HCV were found in this population, I am not sure how much of an impact that will have on the overall, country-wide epidemic. This study certainly supports the case-finding initiatives that are needed for similar settings.

 - General comment: Will this study protocol be registered with a research platform (i.e., clinicaltrials.gov)?

Is the study design appropriate for the research question?

Yes

Is the rationale for, and objectives of, the study clearly described?

Yes

Are sufficient details of the methods provided to allow replication by others?

Yes

Are the datasets clearly presented in a useable and accessible format?

Yes

Reviewer Expertise:

Biostatistics, infectious diseases, public health, viral hepatitis.

I confirm that I have read this submission and believe that I have an appropriate level of expertise to confirm that it is of an acceptable scientific standard.
